# Visualization of the existence of LEAP2 in the nucleus accumbens and its role in amphetamine-induced locomotor activity

**DOI:** 10.1186/s13041-025-01227-5

**Published:** 2025-07-03

**Authors:** Seohyeon Lee, Ga Young Yoo, Hyung Shin Yoon, Jeong-Hoon Kim

**Affiliations:** 1 Department of Medical Sciences, Yonsei University College of Medicine, Seoul, 03722 Republic of Korea; 2 Department of Physiology, Yonsei University College of Medicine, Seoul, 03722 Republic of Korea

**Keywords:** LEAP2, Nucleus accumbens, Amphetamine, Neuropeptide, Locomotor activity

## Abstract

The nucleus accumbens (NAcc) is a key brain region in reward circuitry, mediating responses to psychostimulants, such as amphetamine (AMPH), including locomotor activity. This effect is known to be enhanced by the orexigenic neuropeptide ghrelin acting through growth hormone-secretagogue receptors (GHSR) expressed in the region. Recently, liver-expressed antimicrobial peptide 2 (LEAP2) was identified as another ligand for GHSR that opposes ghrelin’s action. Based on its antagonism, we hypothesized that LEAP2 modulates AMPH-induced locomotor activity in the NAcc. To examine this, we first confirmed the presence of LEAP2 protein in this NAcc and observed that its fluorescent signals were predominantly localized in neurons, including medium spiny neurons (MSNs). We then investigated whether LEAP2 microinjection alters AMPH-induced locomotor activity. Our findings showed that LEAP2 inhibited acute AMPH-induced locomotor activity in a dose-dependent manner. However, its inhibitory effects were absent following chronic AMPH exposure, indicating that the effect of LEAP2 on AMPH-induced locomotor activity varies depending on drug-exposed physiological status. These results provide new insights into a state-dependent regulatory role of LEAP2 in AMPH-induced locomotor activity.

**Supplementary Information:**

The online version contains supplementary material available at 10.1186/s13041-025-01227-5.

## Introduction

The nucleus accumbens (NAcc) is a key neural substrate of the brain’s reward circuitry and plays a central role in mediating rewards induced by both natural stimuli, such as food, and psychostimulants including amphetamine (AMPH) [1]. Upon administration, AMPH increases dopamine levels in the NAcc, eliciting a range of behavioral responses. Among these, the rapid and robust enhancement of locomotor activity serves as a critical indicator for evaluating multiple aspects of drug action, including central nervous system stimulation, neuroplasticity, and addiction-related behavioral alterations [2, 3]. The NAcc expresses a variety of neuropeptides and neuropeptide-related receptors involved in appetite regulation [4], many of which have been consistently reported as modulators of AMPH-induced behavioral responses [5–8]. For example, the cocaine- and amphetamine-regulated transcript (CART) peptide, an anorexigenic neuropeptide, suppresses AMPH-induced locomotor activity when locally injected into the NAcc [7]. Conversely, local infusion of ghrelin, an orexigenic neuropeptide, enhances locomotor activity in response to acute AMPH via the growth hormone secretagogue receptor (GHSR) expressed in this area [6]. These findings highlight the importance of understanding how appetite-related neuropeptides in the NAcc influence AMPH-induced locomotor activity.

Recently, liver-expressed antimicrobial peptide 2 (LEAP2), originally identified for its antimicrobial activity in the liver, has emerged as a novel endogenous ligand of GHSR that acts as an antagonist or inverse agonist [9–11]. LEAP2 also antagonizes ghrelin, which was previously considered the sole ligand for GHSR [12]. Although the role of LEAP2 in AMPH-induced locomotor activity remains largely unexplored, its potential role can be inferred from the established role of ghrelin and GHSR on locomotor responses to psychostimulants such as AMPH and cocaine. For example, increased ghrelin levels, whether induced by food restriction or via direct NAcc infusion, enhance locomotor activity to psychostimulants [6, 13–16]. In contrast, GHSR inactivation through genetic deletion or NAcc-specific pharmacological antagonism inhibits these responses [15, 17]. Considering LEAP2’s antagonistic effect on ghrelin signaling through GHSR, these results suggest that LEAP2 may also modulate psychostimulant-induced locomotor activity.

Based on these findings, we hypothesized that LEAP2 in the NAcc may regulate AMPH-induced locomotor activity. Although LEAP2 expression has been previously reported at the mRNA level in the brain [18], its endogenous protein expression remains unverified. To address this, we first examined whether LEAP2 protein is present in the NAcc. Subsequently, we assessed whether LEAP2 in this region modulates AMPH-induced locomotor activity.

## Materials and methods

### Animals

Male Sprague-Dawley rats weighing 200–230 g (equivalent to 6 weeks old) were obtained from Orient Bio Inc. (Seongnam-si, Korea). They were housed two to three per cage with water and food *ad libitum* under a 12-hour light/dark cycle (lights out at 8:00 p.m.). Rats were acclimatized to their new environment for at least 1 week before experimental procedures began. All animal use procedures were conducted according to an approved Institutional Animal Care and Use Committee protocol of Yonsei University College of Medicine.

### Tissue preparation and immunohistochemistry

Following acclimatization, rats were anesthetized with an intraperitoneal (IP) injection of ketamine (100 mg/kg) and xylazine (6 mg/kg), and were transcardially perfused with 10 mM phosphate buffered saline (PBS, pH 7.4) followed by fixation with 4% paraformaldehyde (PFA). Then, brains were extracted, post-fixed with PFA in 10 mM PBS at 4 °C overnight, and transferred in 10 mM PBS with 30% sucrose and stored at 4 °C. After 2–3 days, brains were embedded in OCT compound mixed with 30% sucrose flash-frozen in chilled isopentane on dry ice. Coronal Sect. (50 μm), ranging from 1.6 to 2.7 mm for the NAcc, -2.2 to -2.8 mm for the hippocampus and the hypothalamus, and − 5.2 to -5.4 mm for the ventral tegmental area, from bregma [19] were obtained using a cryostat. Free-floating sections were blocked in 10 mM PBS (pH 7.4) with 5% normal goat serum (Jackson ImmunoResearch Inc., West Grove, PA, USA) and 0.3% Triton X-100. Then, they were incubated overnight with primary antibodies in 10 mM PBS containing 2% normal goat serum and 0.1% Triton X-100 at 4℃. Sections were washed three times (10 min each) with 10 mM PBS with 0.1% Triton X-100, then incubated with secondary antibodies for 2 h at room temperature. Sections were washed with 10 mM PBS with 0.1% Triton X-100, then mounted on slides with Vectashield mounting medium containing DAPI (Vector Laboratories, Burlingame, CA, USA). The antibodies used were detailed in Table S1. Additional tissues were stained, excluding the primary antibodies, to check for antibody specificity and background noise (data not shown).

### Imaging analysis

All images from the immune-stained brain slices, including the NAcc, were acquired with LSM 700 confocal laser scanning microscope (Carl Zeiss) and analyzed using Zen 3.4 Software (Carl Zeiss, Jena, Germany). The boundaries of the analyzed regions in the image were marked based on the rat brain atlas by Paxinos and Watson [19].

### Cannulation surgery for microinjection

For the behavioral experiments, acclimatized rats underwent surgery for guide cannula implantation into the NAcc. Rats were anesthetized with an IP injection of ketamine (100 mg/kg) and xylazine (6 mg/kg) and the incisor bar was set 5.0 mm above the interaural line. Guide cannulas (22 gauge; Plastics One, Roanoke, VA, USA) were angled at 10 °C to the vertical, and implanted bilaterally into the NAcc 1 mm above the injection site (A/P, + 3.2; L, ± 2.8; D/V, − 6.1 mm from bregma and skull) [20]. Cannulas were secured with dental acrylic cement anchored to stainless steel screws fixed to the skull. Dummy cannulas (28 gauge; Plastics One) extending 1 mm past the tip of the guide cannulas were inserted into the guide cannulas. Meloxicam (1 mg/kg, IP) was administered post-surgery as an analgesic. Rats were allowed to recover for 1 week before behavioral experiments.

### Drugs and microinjection

D-amphetamine-sulfate (U.S. Pharmacopeia, Rockville, MD, USA) was dissolved in saline (sterile 0.9% NaCl) to a concentration of 1 mg/ml. LEAP2 [LEAP2 (37–76) (Rat), Cat. No. 075 − 50, Phoenix Pharmaceuticals Inc., Burlingame, CA, USA] was dissolved in vehicle (sterile 0.9% NaCl) to final concentrations of 0.5 µg/µl or 5.0 µg/µl immediately before use. Bilateral microinjection into the NAcc was done using injection cannulas (28 gauge) connected to 1-µl syringes (Hamilton, Reno, NV, USA) via PE-20 tubing to a depth 1 mm below the guide cannulas. LEAP2 or saline was infused at a volume of 0.5 µl over 30 s. The injection cannulas were left for 1 min to allow the drug to diffuse. The injection volume and timing were optimized to minimize tissue damage and prevent backflow through the syringe, following well-established protocols [21, 22].

### Locomotor activity

Locomotor activity was measured with a bank of nine activity boxes (35 × 25 × 40 cm; IWOO Scientific Corporation, Seoul, Korea) made of translucent Plexiglas. Each box was individually housed in a PVC plastic sound-attenuating cubicle. The floor of each box consisted of 21 stainless steel rods (5 mm diameter) spaced 1.2 cm apart center to center. Two infrared light photo beams (Med Associates, St. Albans, VT, USA) positioned 4.5 cm above the floor and spaced evenly along the longitudinal axis of the box estimated horizontal locomotor activity.

### Extraction of mRNA and quantitative RT-PCR (qRT-PCR)

After behavioral experiments were finished, rats were sacrificed, and brains were rapidly extracted and stored at -80°C. Total RNA was isolated from the NAcc using RNeasy Plus Mini kit (Qiagen, Hilden, Germany) following manufacturer instructions. RNA quantity and quality were assessed with NanoDrop 1000 spectrophotometer (Thermo Fisher Scientific, Waltham, MA, USA). Reverse transcription was performed using the High Capacity cDNA Reverse Transcription Kit (Applied Biosystems, Foster City, CA, USA). qPCR reactions were run on Quantstudio3 (Applied Biosystems) using PowerUp SYBR Green (Applied biosystems) and primer pairs. The following primer pairs were used: Leap2: forward, 5’-GCT CCA ACA TCT TCC TTT TGC C-3’ and reverse, 5’-TCC GGG TTC TCT TTG CTG AA-3’; Ghsr: forward, 5’-GGT GTC CAG CGT CTT CTT CT-3’ and reverse, 5’- GCC ATA GCT TCC TCC CGA TG-3’; Gapdh: forward, 5’-AGG AGT AAG AAA CCC TGG ACC-3’ and reverse, 5’- CAG GCC CCT CCT GTT GTT AT-3’. Each reaction was performed in duplicate. Gene expression was calculated based on the Ct method [23], and Gapdh was used as the reference gene.

### Statistical analysis

Data are presented as standard error of means (+ SEM). Statistical analyses were performed using statistical analysis program of Sigma Plot 12.0. Detailed statistical results were shown in Table S2. After all behavioral experiments, rat brains were removed and sliced at 1 mm intervals to determine the position of the cannula. Only rats with cannula tips located bilaterally in the NAcc were included in the data analysis. Differences between experimental conditions were considered statistically significant when *p* < 0.05.

## Results

### LEAP2 in the NAcc was expressed in neurons including medium spiny neurons (MSN)

**Fig. 1:**
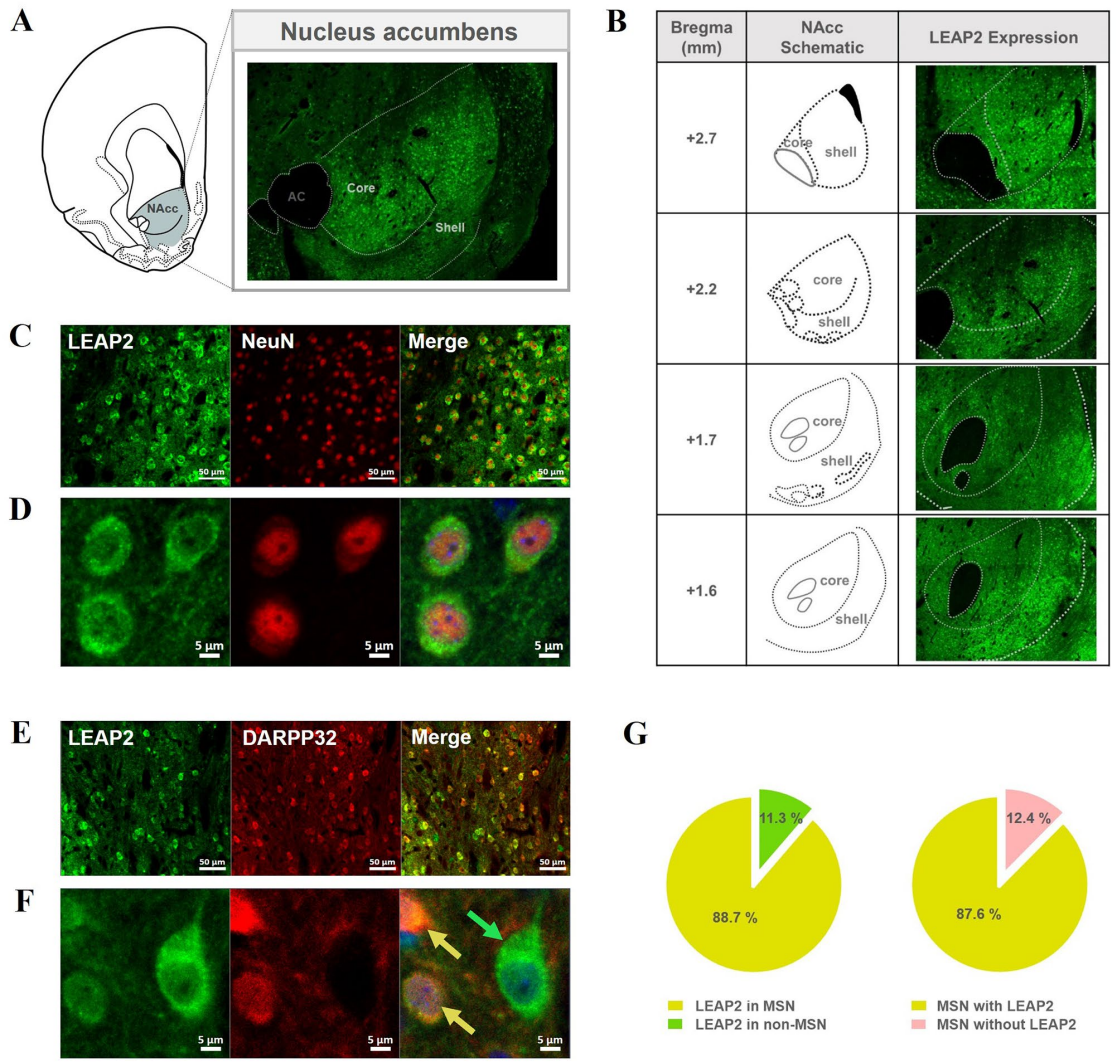
The pattern of LEAP2 expression in the NAcc. (**A**) Fluorescence signals of LEAP2 are shown in the NAcc confirming the existence of LEAP2 at the protein level in this site. The images were taken with 8 × 6 tiles at 20X magnification by confocal microscopy. (**B**) The distribution of LEAP2 expression across different rostro-caudal levels of the NAcc. Images were acquired at 20X magnification as tiled scans, adjusted to match the size of the NAcc at each level (10 × 7, 8 × 6, 10 × 8, and 8 × 7 tiles, respectively). Schematic diagrams illustrate the corresponding rostro-caudal coordinates relative to bregma, delineating the core and shell sub-regions based on the rat brain atlas of Paxinos and Watson [19]. (**C**-**F**) The images were taken at 20X (**C**, **E**; scale bar: 50 μm) or 63X magnification with zoom X3 (**D**, **F**; scale bar: 5 μm) by confocal microscopy. Most LEAP2 fluorescence signals were found to merge with NeuN signals (**C**-**D**). Further, most signals of LEAP2 were also found to merge with the DARPP-32 signal (yellow arrow), while some did not (green arrow) (**E**-**F**). (**G**) Proportion analysis of LEAP2 expression in medium spiny neurons (MSNs) and non-MSNs in the NAcc. The majority of LEAP2-expressing cells were identified as MSNs (co-labeled with DARPP-32, yellow region in the pie chart), while a smaller proportion were non-MSNs (LEAP2-positive but DARPP-32-negative, green region). Within the total MSN population, most MSNs expressed LEAP2 (yellow), whereas a minor fraction of MSNs lacked expression of LEAP2 (pink). Quantification analysis was performed imaging on three naive rats and obtained 6-7 images from each rat, for a total of 19 images

To determine whether LEAP2 is expressed in the NAcc at the protein level, brain sections containing the NAcc obtained from naïve rats were reacted with a LEAP2-specific antibody and analyzed immunohistochemically. LEAP2 fluorescent signals were observed throughout the NAcc (Fig. 1A-B), confirming its expression in this region. These signals were detected in multiple rostro-caudal regions of the NAcc, with greater intensity in the shell compared to the core (Fig. 1B). To examine whether LEAP2 was expressed in neuronal cells, double staining was performed using antibodies against the neuronal marker NeuN and LEAP2. Almost all LEAP2 fluorescence signals were merged with NeuN markers, indicating that most LEAP2 was expressed in neurons in this region (Fig. 1C-D). Since more than 95% of the neurons in the NAcc are known to be MSNs [24], we further checked whether LEAP2 was expressed exclusively in MSNs. To explore this, we determined whether LEAP2 fluorescence signals merged with the MSN marker, dopamine(DA)- and cyclic AMP-regulated neuronal phosphoprotein 32 kDa (DARPP-32; Fig. 1D-E). We found that LEAP2 signals were mostly observed in MSNs (Fig. 1F, yellow arrow), with some expression also detected in non-MSNs (Fig. 1F, green arrow). 

For quantitative analysis of LEAP2 and DARPP-32 co-expression, we examined 3–4 slices per animal (*n* = 3) and analyzed a total of 19 images from the core and shell regions (Fig. 1G, Table S3). Approximately 89% of LEAP2-expressing cells were MSNs, as indicated by co-labeling with DARPP-32, while the remaining cells were non-MSNs. Among the total number of DARPP-32-labelled MSN cells, about 88% expressed LEAP2. Since MSNs express dopamine receptors [25], which play a critical role in psychostimulant-induced behaviors, we further examined the co-expression of LEAP2 with dopamine receptor subtypes, dopamine receptor 1 (D1R) and 2 (D2R). Our results revealed that LEAP2 is co-localized with both D1R- and D2R-expressing MSNs, with approximately 72% of D1R-positive neurons and 57% of D2R-positive neurons expressing LEAP2, indicating relatively higher co-expression in D1R-MSNs compared to D2R-MSNs (Figure S1).

Additionally, we also observed the neuron-specific expression of LEAP2 in several brain regions, including the hippocampus, the hypothalamus, and the ventral tegmental area, which are involved in appetite- and reward-related behaviors and form synaptic connections with the NAcc (Figure S2). Similar to the NAcc, the majority of LEAP2 fluorescent signals in these three regions colocalized with the neuron marker NeuN.

### Microinjection of LEAP2 into the NAcc inhibited acute but not chronic AMPH-induced locomotor activity

**Fig. 2:**
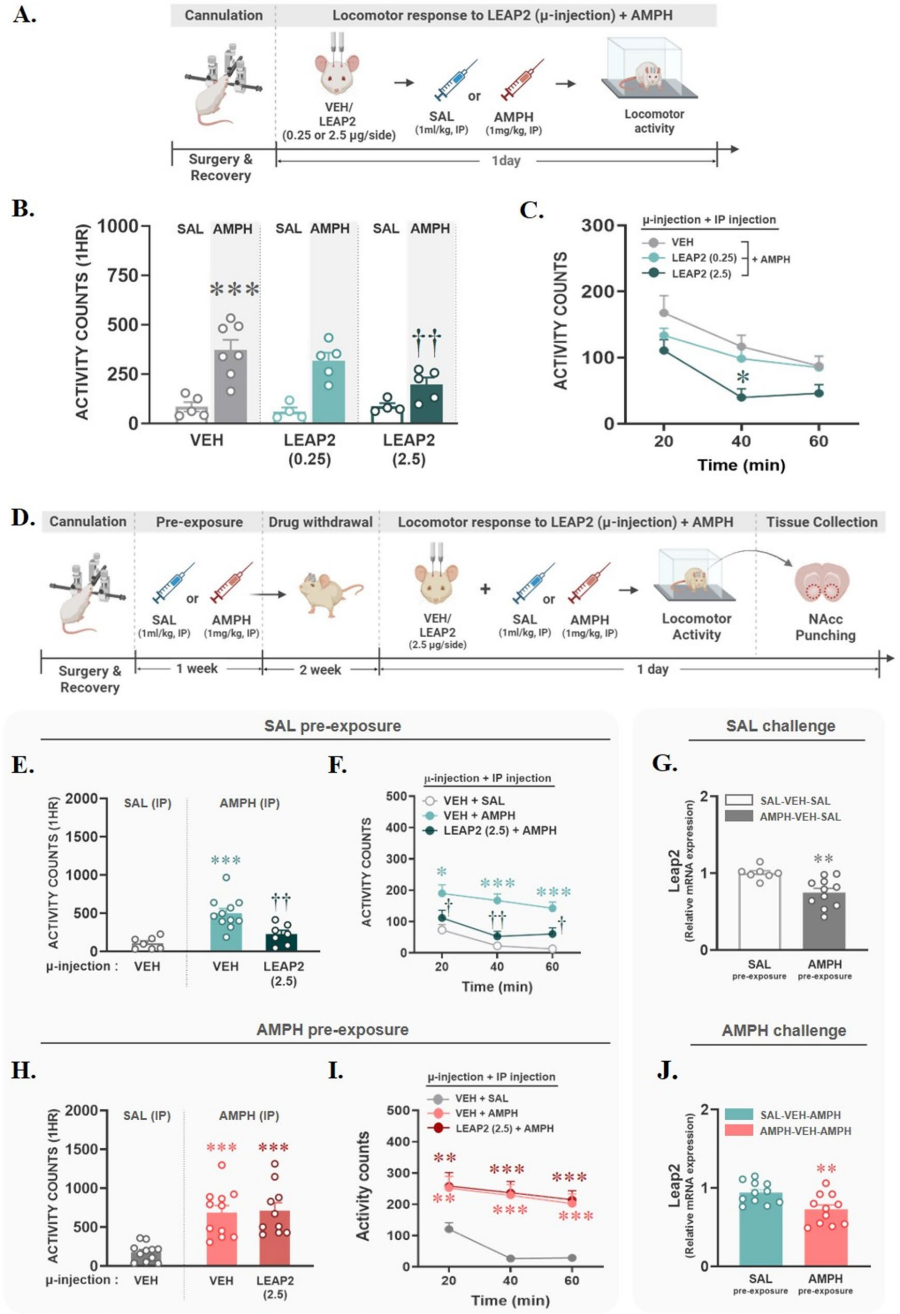
LEAP2 microinjection into the NAcc blocked acute, but not chronic, AMPH-induced locomotor activity in a dose-dependent manner. (**A**) The experimental procedures to explore the role of LEAP2 on acute AMPH-induced locomotion are depicted. (**B**-**C**) After cannulation surgery and recovery, rats were microinjected with vehicle (VEH) or LEAP2 (0.25–2.5 µg/side) into the NAcc, followed by systemic injection of saline (SAL) or AMPH. Then, their locomotor activity was measured for 1 h, with results shown as mean + SEM of 1-hour total score (**B**, ***p < 0.001, vs. VEH-SAL; ^††^*p* < 0.01, vs. VEH-AMPH; two-way ANOVA by post-hoc Tukey) and time course (**C**, *p < 0.05, vs. 40 min of VEH-AMPH; one-way RM ANOVA by post-hoc Tukey). (**D**) The experimental procedures to explore the role of LEAP2 on chronic AMPH-induced locomotion are depicted. (**E**-**F**, **H**-**I**) Rats exposed to chronic SAL or AMPH after cannulation surgery and recovery were microinjected with VEH or LEAP2 (2.5 µg/side) into the NAcc, followed by systemic injection of SAL or AMPH. Then, their locomotor activity was measured for 1 h, with results shown as mean + SEM of 1-hour total score (**E**, **H**) and time course (**F**, **I**). In SAL pre-exposure, LEAP2 blocked hyperlocomotion induced by the acute AMPH (**E**, ***p < 0.001, vs. VEH-SAL; ^††^*p* < 0.01, vs. VEH-AMPH; one-way ANOVA by post-hoc Tukey). However, the inhibitory role of LEAP2 was disappeared by AMPH pre-exposures (**H**, ***p < 0.001, vs. VEH-SAL; one-way ANOVA by post-hoc Tukey) and time course (**I**, **p < 0.01, vs. 20-min of VEH-SAL; ***p < 0.001, vs. 40–60 min of VEH-SAL; one-way RM ANOVA with post-hoc Tukey). (**G**, **J**) LEAP2 mRNA expression in the NAcc was significantly reduced by AMPH pre-exposure, independent of the challenge condition (**p < 0.01, vs. SAL pre-exposure; t-test). Created with BioRender.com

To investigate the role of LEAP2 in the NAcc in AMPH-induced locomotor activity, all rats were subjected to cannulation surgery in the NAcc. After a 1-week recovery period, rats were habituated in activity boxes for 1 h, and then they were microinjected bilaterally with vehicle (VEH) or one of two doses of LEAP2 (0.25–2.5 µg/side). Immediately after microinjection, saline (SAL, 1 ml/kg) or AMPH (1 mg/kg) was administered via IP injection, and the locomotor activity of each group was measured for 1 h. All experimental procedures are illustrated in Fig. 2A. Microinjection of LEAP2 in the NAcc did not alter locomotor activity in the SAL group, confirming that the two doses of LEAP2 alone did not alter baseline locomotion. However, LEAP2 reduced AMPH-induced hyperlocomotion at the dose of 2.5 µg/side (*p* < 0.01; Fig. 2B), most significantly at the 40-min time point after AMPH injection (*p* < 0.05; Fig. 2C). To determine the role of LEAP2 in hyperlocomotion following repeated exposure to AMPH, all rats underwent cannulation surgery and were administered SAL or AMPH (1 mg/kg, IP) four times, 2–3 days apart. After a 2-week withdrawal period, they were then microinjected with VEH or LEAP2 (2.5 µg/side), followed by a challenge with SAL (1 ml/kg, IP) or AMPH (1 mg/kg, IP). Locomotion was measured for 1 h. These experimental procedures are illustrated in Fig. 2D. The inhibitory effect of LEAP2 was confirmed even when rats were given a challenge injection of AMPH following repeated SAL pre-exposure (*p* < 0.01, vs. SAL-VEH-AMPH; Fig. 2E), and this effect was observed at all time points after AMPH injection (*p* < 0.05; vs. 20- or 60-min time points; *p* < 0.01, vs. 40-min time point in SAL-VEH-AMPH; Fig. 2F). However, unlike its inhibitory effect on acute AMPH, LEAP2 failed to block hyperlocomotion induced by chronic AMPH pre-exposure (Fig. 2H), and there were no significant differences compared to the VEH-AMPH group at any measured time point (Fig. 2I). To determine whether the functional changes in LEAP2 caused by chronic AMPH pre-exposure are associated with changes in its expression in the NAcc, the NAcc was extracted from four groups that had not received LEAP2 microinjections: SAL-VEH-SAL, SAL-VEH-AMPH, AMPH-VEH-SAL, and AMPH-VEH-AMPH. Then, LEAP2 and its receptor, GHSR, expression was analyzed using qRT-PCR. As a result, LEAP2 mRNA expression in the NAcc was significantly reduced by AMPH pre-exposure (*p* < 0.01), and this reduction persisted regardless of whether the final challenge drug was SAL (Fig. 2G) or AMPH (Fig. 2J). However, unlike in LEAP2, no significant differences in GHSR mRNA expression were found between groups (Figure S3). These findings indicate that the impact of chronic AMPH exposure on LEAP2 expression is long-lasting and may contribute to functional changes in LEAP2’s role.

## Discussion

We visualized the expression of LEAP2 in the NAcc and investigated its effect on the locomotor activity of AMPH. To the best of our knowledge, it is the first demonstration of LEAP2 expression at the protein level and its role on psychostimulant drugs in this site.

We first confirmed that LEAP2 is expressed in neurons within the NAcc, predominantly in medium spiny neurons (MSNs). While the release mechanism of LEAP2 is not yet well understood, neuropeptides are generally secreted at synaptic terminals and act on postsynaptic sites, similar to classical neurotransmitters. Moreover, neuropeptides exhibit hormone-like properties, influencing targets distal from the site of release [26, 27]. Given that MSNs project to regions such as the ventral pallidum, lateral hypothalamus, and ventral tegmental area [28], LEAP2 may influence downstream targets via these established pathways. Nevertheless, local effects within the NAcc or actions on non-projection sites cannot be ruled out.

We have previously shown that approximately 75–90% of MSNs in the NAcc express GHSR [29]. This aligns with the expression profile of LEAP2 observed in this study, suggesting that some MSNs may express both GHSR and LEAP2. Based on this anatomical evidence, LEAP2 may modulate GHSR-expressing neurons either within the same cell or through local interactions with nearby neurons, although the precise mode of action remains to be determined.

Since MSNs in the NAcc also express dopamine receptors [25], the presence of LEAP2 in these neurons suggests a potential involvement in dopaminergic signaling. Supporting this notion, our colocalization analysis (Figure S1) revealed that LEAP2 is expressed in both D1R- and D2R-expressing MSNs, providing anatomical evidence for a possible interaction with dopamine pathways. Although direct evidence for LEAP2’s role in dopaminergic modulation within the NAcc is still lacking, prior studies provide relevant insights. For instance, in the hippocampus, GHSR has been shown to form heterodimers with dopamine receptors to modulate dopaminergic signaling [30], and in HEK293T cells, LEAP2 stabilizes a GHSR–D2R complex, thereby modulating dopamine signaling [31]. More recently, centrally administered LEAP2 has been shown to reduce DA release into the NAcc [11]. Taken together, these findings suggest a potential link between LEAP2 and dopaminergic modulation via GHSR, warranting the need for further investigation in the NAcc.

Psychostimulants, including AMPH, are known to increase DA levels in the NAcc, and the addictive behaviors resulting from repeated exposure to these substances are mediated through changes in DA signaling in this region [32]. Our results showed that LEAP2 inhibited acute AMPH-induced locomotor activity in a dose-dependent manner, but this effect was not observed after chronic AMPH exposure, suggesting its state-dependent neuropeptidergic modulation. Recent finding has shown that centrally injected LEAP2 can regulate DA signaling by attenuating the rapid increase of DA in the NAcc induced by alcohol [18]. Similarly, it is raising the possibility that LEAP2 may influence the dopaminergic responses to acute AMPH, either directly or indirectly.

However, our findings also indicate that this modulatory effect of LEAP2 is abolished following chronic AMPH exposure. The diminished effect of LEAP2 following chronic AMPH exposure could be attributed to several changes in the NAcc. First, chronic AMPH is known to alter DA signaling sensitivity and receptor responsiveness [33], which may reduce LEAP2’s modulatory capacity. Second, our data show that LEAP2 mRNA expression in the NAcc was significantly reduced following chronic AMPH exposure, whereas GHSR expression remained unchanged (Figure S3). Additionally, our separate experiment (manuscript in preparation) demonstrated that chronic AMPH exposure alters responsiveness to endogenous ghrelin, suggesting a disruption in the local balance of GHSR signaling that may contribute to the altered function of LEAP2. Third, AMPH has been reported to affect the expression of various neuropeptides [34], raising the possibility that altered peptide interactions may influence LEAP2 function. Overall, while the precise mechanisms remain to be fully elucidated, these observations suggest that changes in DA signaling, local balance of GHSR signaling, or broader neuropeptidergic interactions may collectively contribute to the altered behavioral effects of LEAP2 following chronic AMPH exposure.

In addition to these neurochemical changes, transcriptional regulation of LEAP2 may also contribute to its diminished function following chronic AMPH exposure. Our results indicate that acute AMPH exposure does not alter LEAP2 mRNA. In contrast, chronic AMPH exposure followed by a two-week abstinence led to a significant reduction in LEAP2 mRNA expression, which remained suppressed even after AMPH re-exposure, indicating a lasting transcriptional adaptation. Previous studies have shown that repeated exposure to cocaine, a psychostimulant similar to AMPH, can lead to sustained downregulation of specific genes lasting up to 100 days during abstinence [35]. While the report has focused on withdrawal states, our findings extend this understanding by demonstrating that such gene expression changes can persist even after drug re-exposure. Taken together, sustained LEAP2 mRNA downregulation may reduce peptide levels in the NAcc, weakening its neuro-regulatory role and contributing to the loss of efficacy after chronic AMPH exposure. To further elucidate this transcriptional adaptation, a time course analysis of LEAP2 mRNA expression across different stages of AMPH exposure, including acute administration, repeated exposure, various withdrawal intervals, and post-reinstatement, would be highly informative. This approach could clarify the temporal progression and potential reversibility of LEAP2 suppression and help distinguish transient responses from long-term transcriptional adaptations relevant to addiction pathology.

To the best of our knowledge, our study is the first to demonstrate that LEAP2 in the NAcc can regulate locomotor activity to psychostimulants, revealing a novel role for LEAP2 in modulating reward-related neural circuits. However, several limitations should be considered when interpreting these findings. First, to detect LEAP2 protein in brain tissue, we used a commercially available polyclonal antibody, which has been previously validated for specificity and sensitivity [36]. While these validations support the antibody’s reliability, future studies employing genetic models (e.g., LEAP2 knockout or overexpression) would help further confirm its specificity in neural tissue. Second, while this study offers an initial indication of LEAP2’s involvement in psychostimulant-induced behaviors, a more comprehensive behavioral analysis is essential to fully elucidate its regulatory role in addiction. Future research should explore LEAP2’s effects across a broader spectrum of addiction-related paradigms, including conditioned place preference and self-administration models. Lastly, the current study was conducted exclusively in male rats. Recent findings indicate that there exist sexual-dimorphisms in the role of LEAP2 and its receptors [11, 37, 38]. Therefore, it remains to be determined whether it is the case in the function of LEAP2 in AMPH-induced behavior, as demonstrated in this study.

## Electronic supplementary material

Below is the link to the electronic supplementary material.

Supplementary Material 1

## Data Availability

No datasets were generated or analysed during the current study.

## Abbreviations

LEAP2: Liver-expressed antimicrobial peptide 2
GHSR: Growth-hormone secretagogue receptor
NAcc: Nucleus accumbens
CART: Cocaine- and amphetamine-regulated transcript
MSN: Medium spiny neuron
DARPP32: Dopamine- and cyclic-AMP-regulated phosphoprotein 32 kDa
IP: Intraperitoneal
SAL: Saline
AMPH: Amphetamine
VEH: Vehicle
RT-qPCR: Reverse transcription quantitative polymerase chain reaction
D1R: Dopamine receptor 1
D2R: Dopamine receptor 2
