# Supplementary material for: Visualization of the existence of LEAP2 in the nucleus accumbens and its role in amphetamine-induced locomotor activity
Source: Mol Brain. 2025 Jul 3;18:57. doi: 10.1186/s13041-025-01227-5 (PMC12224806; doi:10.1186/s13041-025-01227-5)
Supplement: Supplementary file 1 — Supplementary Material 1 [file 13041_2025_1227_MOESM1_ESM.docx]

**Supplement**

**Visualization of the existence of LEAP2 in the nucleus accumbens and its role in amphetamine-induced locomotor activity**

Lee et al.

**This PDF file includes:**

Table S1, S2, S3

Figure S1, S2, S3

**Table S1.** Information about the antibody and dilution ratios used for immunostaining experiments to detect LEAP2 expression.


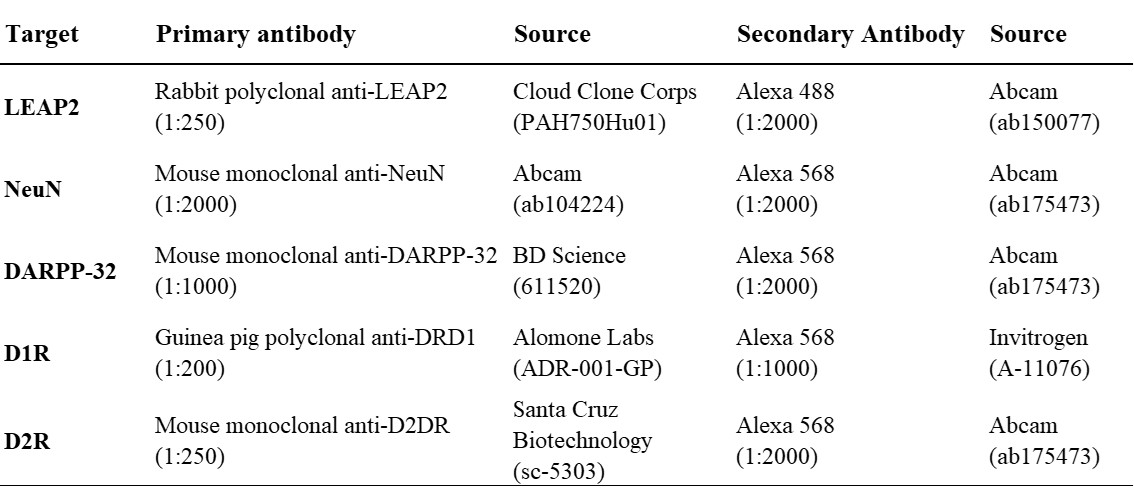


**Table S2.** Statistical methods and results used in Fig. 2


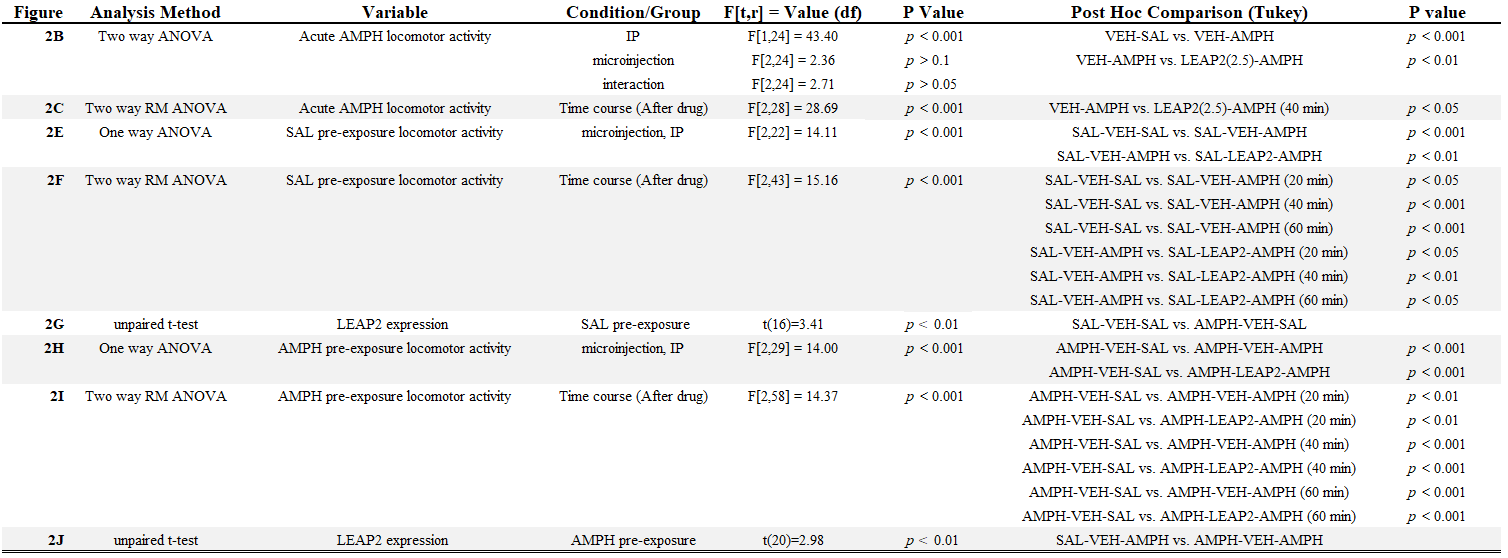


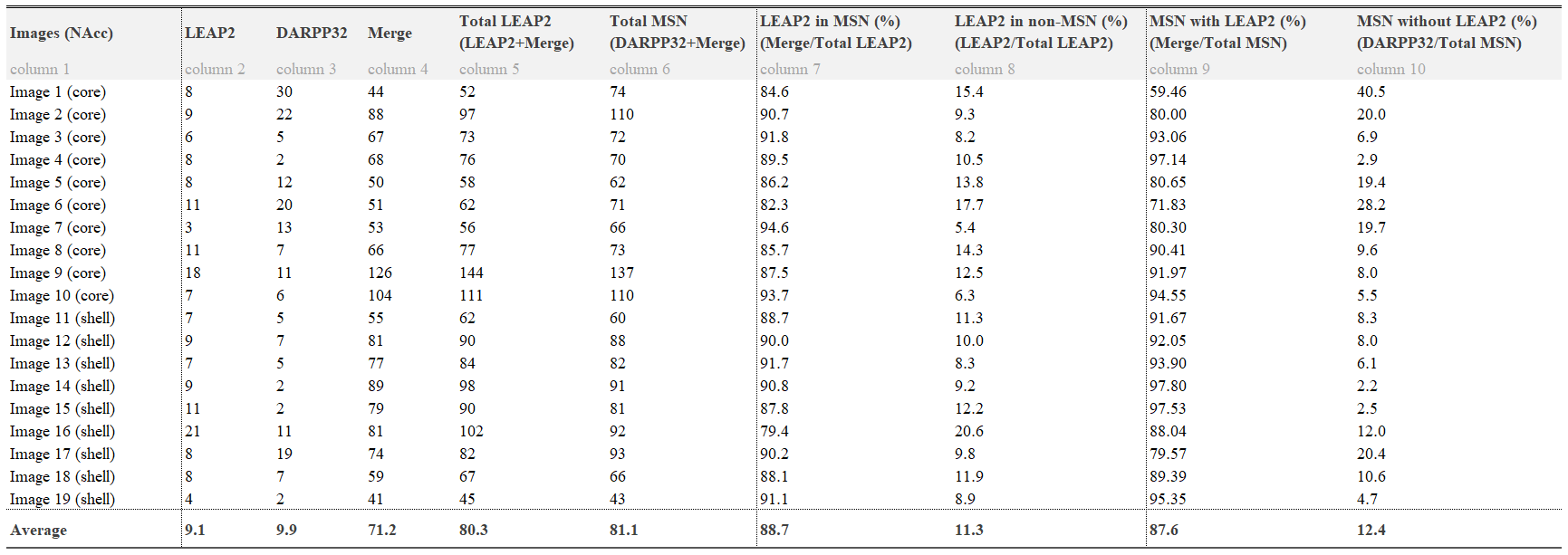
**Table S3.** Summary of cell counts from MSNs.

**Figure S1**

**
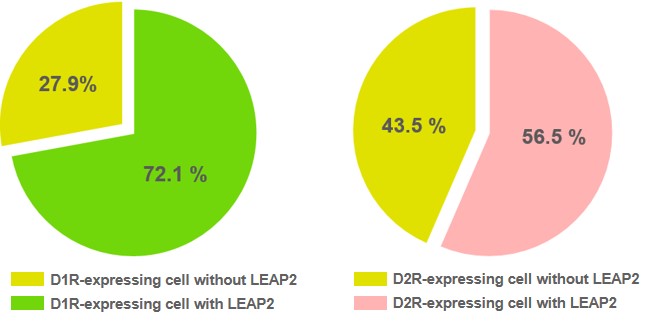

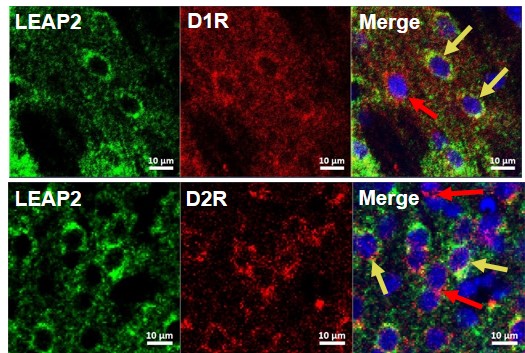
A. B. B.**


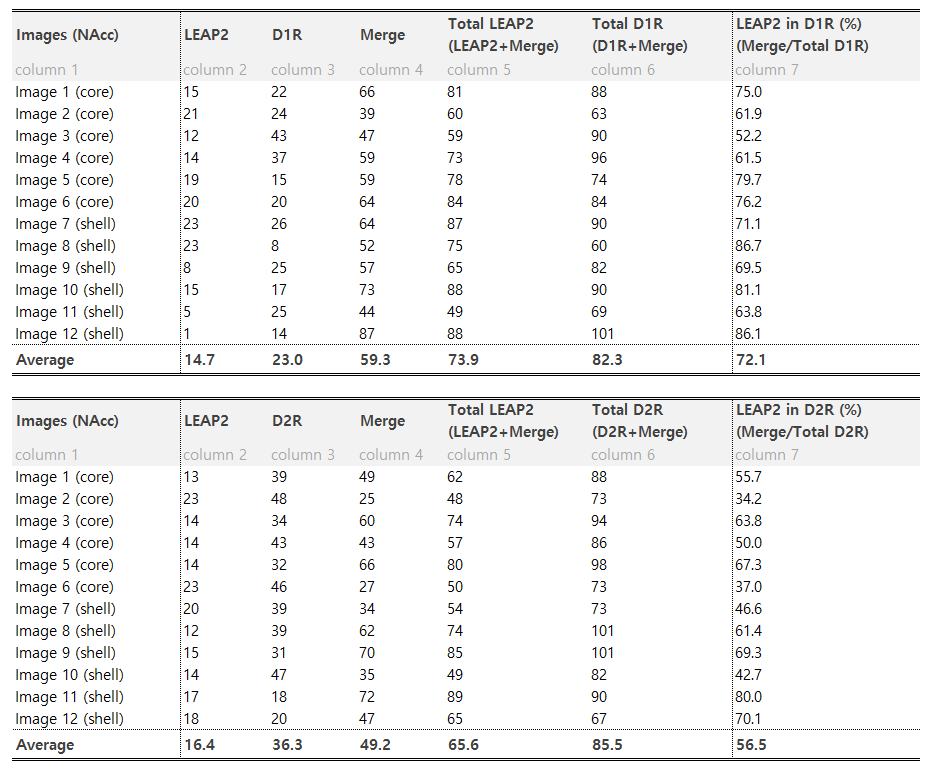
**C.**

**Figure S1. The proportion of LEAP2-expressing cells within D1R- positive and D2R-positive neuron populations in the NAcc**. **(A)** Magnified representative area from an image taken at 20× magnification (red arrow: D1R only; yellow arrow: D1R+LEAP2 merge). **(B)** In D1R-positive cells, 72.1% co-expressed LEAP2 (green region), whereas in D2R-positive cells, 56.5% co-expressed LEAP2 (pink region). These results indicate that LEAP2 expression is more prominent in D1R-positive cells than in D2R positive cells. **(C)** Quantitative analysis was performed imaging on three naive rats and obtained 4 images from each rat, for a total of 12 images, respectively.

**Figure S2**

**
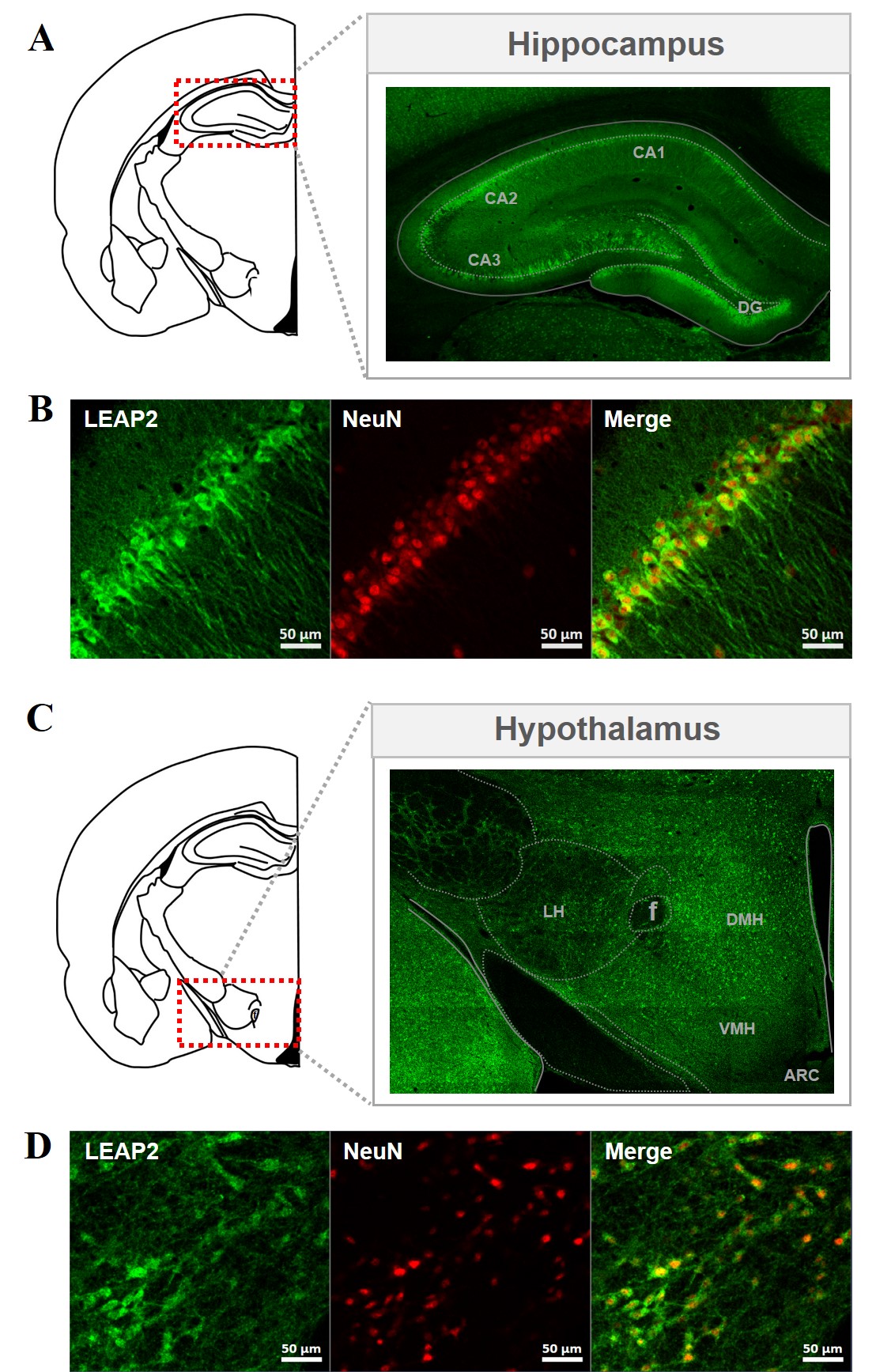
**

**
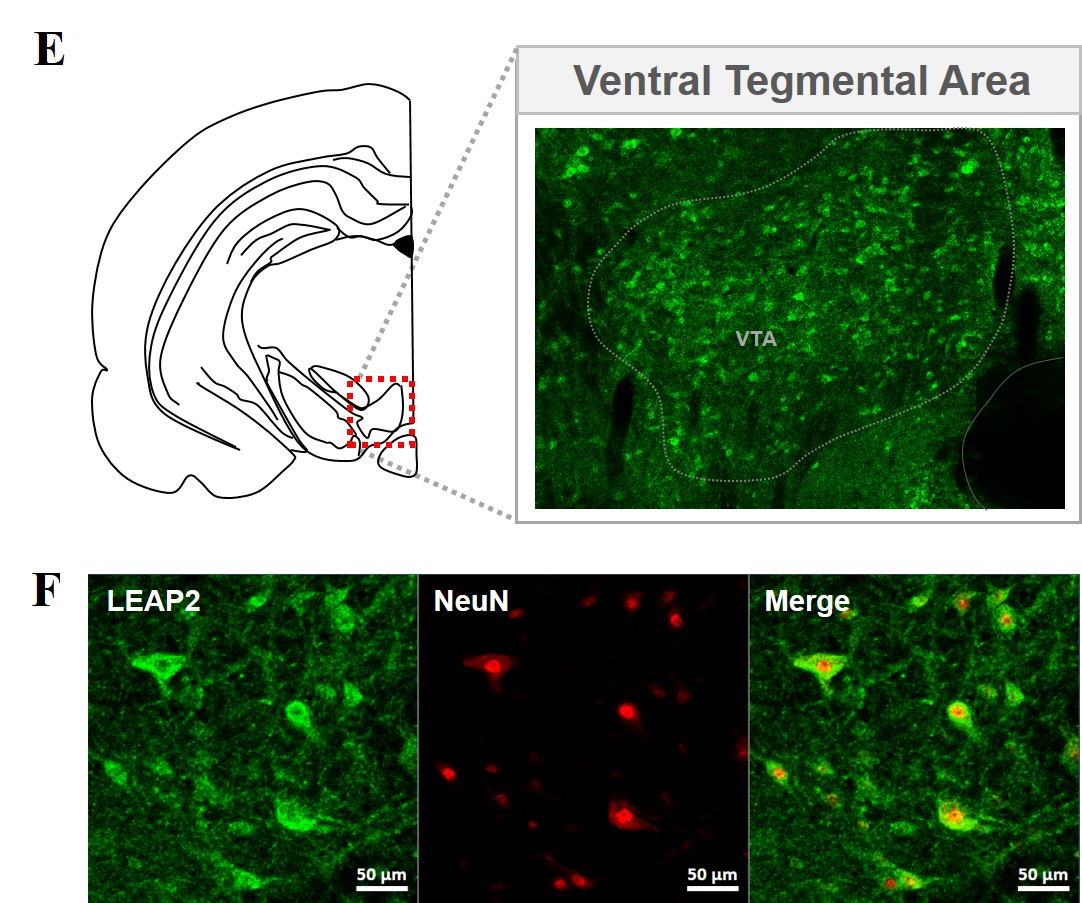
**

**Figure S2**. **LEAP2 fluorescence signals in the hippocampus (A-B), the hypothalamus (C-D), and the ventral tegmental area (E-F).** **(A, C, E)** Fluorescence signals of LEAP2 are shown in the hippocampus (A), the hypothalamus (C), and around the ventral tegmental area (E). These images were taken using a confocal microscope at 20X magnification, with the number of tiles sufficient to encompass each region. **(B, D, F)** LEAP2 fluorescence signals were observed to merge with the neuron marker NeuN, suggesting that almost all of LEAP2 in these regions is expressed in neurons. Images of LEAP2 expression in the CA1 area of the hippocampus (B), the lateral hypothalamus around the fornix (D) and the ventral tegmental area (F) were taken with a confocal microscope at 20x magnification (scale bar: 50 μm); **CA:** cornu ammonis; **DG:** dentate gyrus; **LH:** lateral hypothalamus; **DMH:** dorsomedial hypothalamus; **VMH:** ventromedial hypothalamus; **Arc:** arcuate nucleus; **f**: fornix; **SN**: substantia nigra; **VTA:** ventral tegmental area.

**Figure S3**

**A B**

**
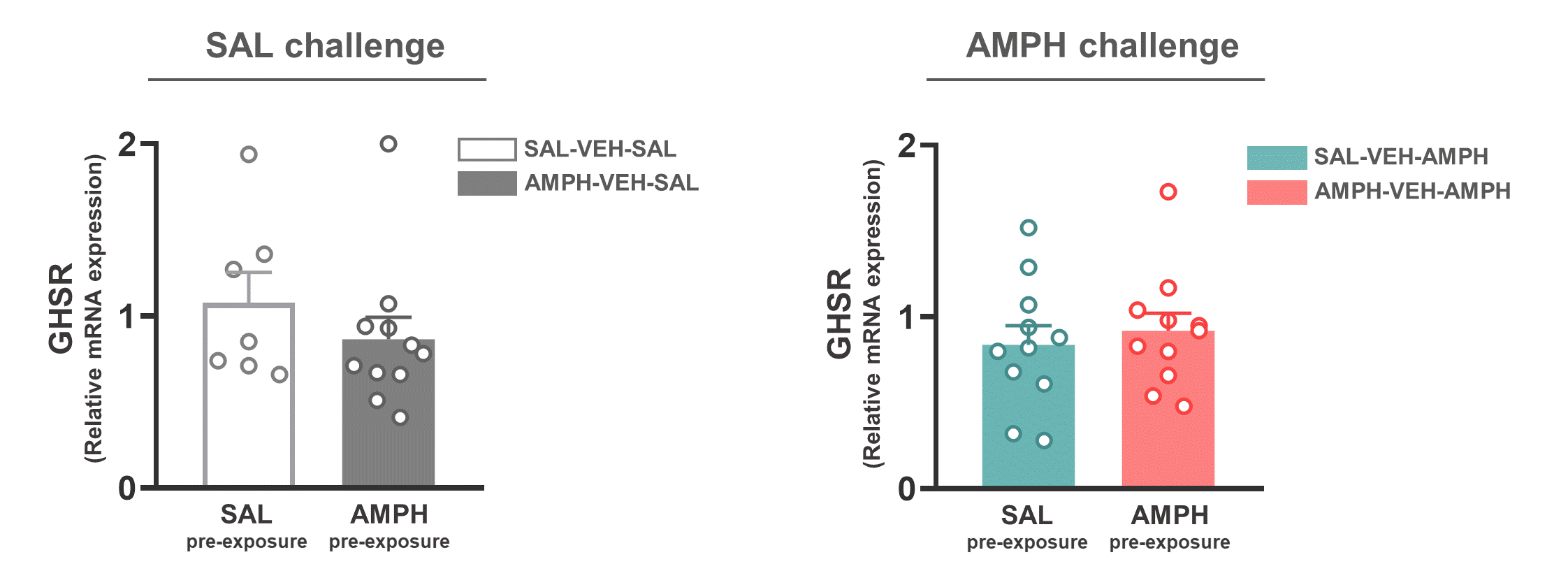
**

**Figure S3. Relative GHSR mRNA expression in the NAcc following AMPH pre-exposure for SAL or AMPH challenge conditions, measured by qRT-PCR.** No significant differences in GHSR expression were observed between SAL- and AMPH-pre-exposed groups under either challenge condition. **(A)** SAL challenge: *p* = 0.34 vs. SAL pre-exposure; **(B)** AMPH challenge: *p* = 0.60 vs. SAL pre-exposure; unpaired t-test. Data are presented as mean + SEM.
